# Preparation and Properties of High Sound-Absorbing Porous Ceramics Reinforced by In Situ Mullite Whisker from Construction Waste

**DOI:** 10.3390/molecules29143419

**Published:** 2024-07-21

**Authors:** Kaihui Hua, Xiaobing Chen, Anze Shui, Xiuan Xi, Pinhai Gao, Yu Zheng, Chuncan He

**Affiliations:** 1 School of Environment and Civil Engineering, Dongguan University of Technology, Dongguan 523808, China; huakh@dgut.edu.cn (K.H.); chen_xb4869@163.com (X.C.); gaoph@dgut.edu.cn (P.G.); zhengy@dgut.edu.cn (Y.Z.); hechuncan@dgut.edu.cn (C.H.); 2 School of Materials Science and Engineering, South China University of Technology, Guangzhou 510641, China; 3 Guangdong Provincial Key Laboratory of Intelligent Disaster Prevention and Emergency Technologies for Urban Lifeline Engineering, Dongguan 523808, China; 4 School of Physical Sciences, Great Bay University, Dongguan 523000, China

**Keywords:** construction waste, porous ceramics, whisker skeleton, sound-absorbing, reinforce

## Abstract

Porous sound absorption ceramic is one of the most promising materials for effectively eliminating noise pollution. However, its high production cost and low mechanical strength limit its practical applications. In this work, low-cost and in situ mullite whisker-reinforced porous sound-absorbing ceramics were prepared using recyclable construction waste and Al_2_O_3_ powder as the main raw materials, and AlF_3_ and CeO_2_ as the additives, respectively. The effects of CeO_2_ content, AlF_3_ content, and sintering temperature on the microstructure and properties of the porous ceramics were systematically investigated. The results showed that a small amount of CeO_2_ significantly promoted the growth of elongated mullite crystals in the resultant porous ceramics, decreased the growth temperature of the mullite whiskers, and significantly increased the biaxial flexural strength. When 2 wt.% CeO_2_ and 12 wt.% AlF_3_ were added to the system, mullite whiskers were successfully obtained at a sintering temperature of 1300 °C for 1 h, which exhibited excellent properties, including an open porosity of 56.4 ± 0.6%, an average pore size of 1.32–2.54 μm, a biaxial flexural strength of 23.7 ± 0.9 MPa, and a sound absorption coefficient of >0.8 at 800–4000 Hz.

## 1. Introduction

Due to rapid industrial development, noise pollution has become a major environmental concern. The use of porous sound absorption materials is considered an effective approach to eliminating noise pollution. Generally, sound-absorbing materials include organic polymer porous materials, metallic porous materials, and inorganic porous materials [1,2,3]. Organic materials are flammable and prone to aging. Metallic materials are expensive and have poor corrosion resistance. The inorganic porous materials [4,5] have excellent properties, such as fire resistance, corrosion resistance, anti-moth properties, and high-porosity. Porous ceramics have received considerable widespread attention, owing to their unique properties. Among the various porous ceramics, porous anorthite-mullite-corundum ceramics [6] have excellent performance characteristics, such as greater high-temperature creep strength, a higher softening point, and a lower expansion coefficient [7], making them promising sound absorption materials [8]. He et al. [3] prepared high-strength porous ceramics by growing magnesium borate whiskers in situ. They found that as whisker content increased, the absorption curve shifted to the low-frequency direction. The absorption coefficient gradually increased, and the absorption coefficient reached 0.58 in the frequency range of 200–1400 Hz. Chen et al. [9] prepared a ceramic foam using a pore-forming agent with a porosity of > 80% and a maximum sound absorption coefficient of 0.86 in the sound frequency range of 200–4000 Hz. Du et al. [10] used a mixture of surfactants to prepare high-porosity SiO_2_ ceramics through direct foaming. The resulting foam ceramics had porosities ranging from 84.61 to 91.35%, compressive strengths ranging from 5.89 to 0.94 MPa, and a maximum acoustic absorption coefficient of 0.69 at the frequency range of 1000–4000 Hz. Generally, kaolin clay [11], feldspar [12], and quartz have been used as the main raw materials to fabricate porous anorthite-mullite-corundum ceramics. However, they have a limited reserve, and are expensive. As a result, researchers have sought new types of abundant and low-cost minerals to replace the starting materials, such as kaolinite gangue [13], construction waste [6,14], and fly ash [15,16]. Among them, construction waste is mainly generated from inorganic non-metallic materials, such as mud and residue generated in the process of building construction, building demolition, and building decoration. Construction waste is produced in large quantities and has a low utilization rate, resulting in various environmental issues [17]. It is rich in alumina, silicon oxide, and similar minerals. As a result, it can be used as the starting material to fabricate porous anorthite-mullite-corundum ceramics, thereby solving the above environmental problems.

For effective sound absorption, the sound absorption materials should have an appropriate open porosity with numerous fine, uniform, and connected pores. Porous ceramics with whiskers or fibers meet these criteria [18,19]. Specifically, the in situ fabricated whisker-reinforced porous ceramics characterized by high open porosities and abundant whisker skeletons are highly effective in obstructing the soundwave and converting the sound energy to thermal energy. Furthermore, the whisker skeletons also enhance the strength of the porous materials [20]. Whisker catalysts (including H_12_AlF_6_N_3_ [13], AlF_3_ [6], NH_4_F [21], and other fluoride additives) and mineralizing agents (including V_2_O_5_ [22], MoO_3_ [23], WO_3_ [24], and CeO_2_ [25]) are used to fabricate the in situ mullite whiskers. Among them, CeO_2_, a rare earth oxide, is used as a high melting point stable additive, and it consistently enhances the mullitization behavior and morphology during the fabrication of mullite whiskers [26,27].

In recent years, preparing porous mullite ceramics using inexpensive industrial wastes to minimize raw material expenses and overall energy consumption has attracted considerable attention from researchers. Wang et al. [20] prepared low-cost porous acoustic ceramics using steel slag and kaolin as raw materials, and V_2_O_5_ and AlF_3_ as additives to enhance the secondary mullitization reaction at reduced temperatures. The prepared ceramics exhibited impressive characteristics, including an open porosity of 65% and a compressive strength of 6.8 MPa. Huo et al. [28] prepared high-strength mullite whisker porous ceramics by calcining high-alumina fly ash as raw material at 1300 °C. The samples had a high-porosity of 49% and a compressive strength of 13 MPa. Liu and Xiang [29] prepared porous mullite ceramics using photovoltaic silicon waste as the raw material, and ammonium molybdate tetrahydrate as the additive. These materials reduced the reaction temperature of mullitization to 200 °C, and were sintered at 900 °C. The porous mullite ceramics exhibited pore diameters of 0.23 μm and 0.5 μm. Furthermore, these ceramics exhibited a flexural strength of 52.8 MPa, and a porosity of 41.8%.

In this work, CeO_2_ was proposed and used as the nucleation agent. Mullite whisker-reinforced porous sound-absorbing ceramics were produced using construction waste and Al_2_O_3_ powder as the main raw materials, and AlF_3_ as the crystallization catalyst. The effects of CeO_2_, AlF_3_, and sintering temperature on the microstructure and properties of the porous ceramics were systematically investigated. The formation and growth mechanism of the mullite whiskers is also discussed in detail.

## 2. Results and Discussions

**Figure 1:**
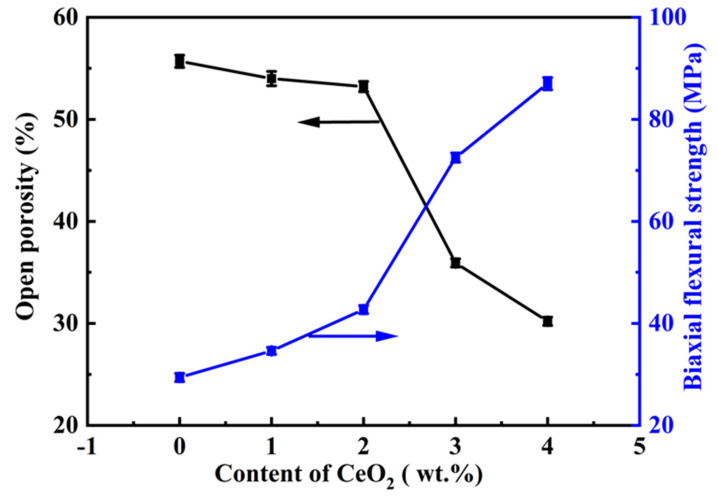
Effect of CeO_2_ concentrations on the open porosity and biaxial flexural strength of the specimens.

Figure 1 illustrates the influence of CeO_2_ concentration on the open porosity and biaxial flexural strength of the samples containing 12 wt.% AlF_3_. As illustrated in Figure 1, as the concentration of CeO_2_ increased, the open porosity of the samples decreased, whereas the biaxial flexural strength was enhanced. Meanwhile, a small amount of CeO_2_ (0–2 wt.%) significantly enhanced the biaxial flexural strength and slightly reduced the open porosity. As the CeO_2_ content increased from 0 to 2 wt.%, the biaxial flexural strength improved by 13.3 MPa, increasing from 29.4 ± 0.8 to 42.7 ± 0.8 MPa, while the open porosity decreased from 55.7 ± 0.6% to 53.2 ± 0.5%, decreasing by 2.5 ± 0.6%. When the content of CeO_2_ was further increased, sharp decreases and increases were observed in both the biaxial flexural strength and the open porosity, respectively.

**Figure 2:**
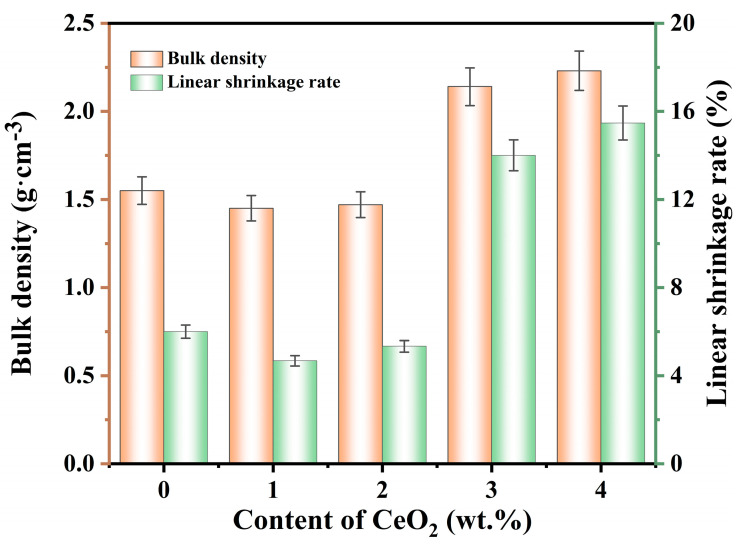
Effect of CeO_2_ concentrations on the bulk density and linear shrinkage rate of the specimens.

Figure 2 illustrates the bulk density and linear shrinkage rate of specimens containing 12 wt.% AlF_3_ and different quantities of CeO_2_ (0–4 wt.%). As the CeO_2_ content increased, the bulk density and linear shrinkage of the samples slightly decreased in a certain range (0–2 wt.%), and then increased (2–4 wt.%). This phenomenon occurred because a small amount of CeO_2_ promoted the mullitization reaction of Al_2_O_3_ and SiO_2_, resulting in the production of more mullite whiskers. Compared to spherical or massive particles, the voids between the whiskers were larger, making the filling effect less uniform, and reducing overall bulk density. The uniform distribution of mullite whiskers prevented uneven local shrinkage and stress concentration in porous ceramics, thereby reducing the fluctuation of linear shrinkage. The specimen with 2 wt.% CeO_2_ exhibited the lowest bulk density (1.47 g·cm^−3^) and lower linear shrinkage (5.33%). Excess CeO_2_ contributed to the formation of a glassy phase in the ceramic materials. The glassy phase had high mobility and could fill the voids and micropores in the ceramic materials during sintering, increasing the bulk density of the ceramics. The glass phase exhibited certain deformation and plasticity, allowing it to flow and deform at high temperatures to fill the microscopic pores during the sintering process of the ceramic material. These deformation and plasticity properties increased the overall linear shrinkage of the ceramic material.

**Figure 3:**
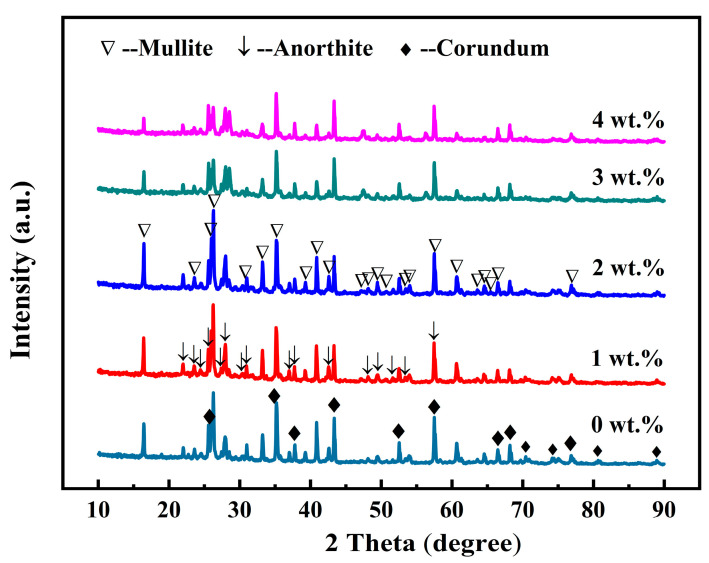
X-ray diffraction patterns of the porous ceramics with different content of CeO_2_.

Figure 3 illustrates the X-ray diffraction (XRD) patterns of the samples containing 12 wt.% AlF_3_ and varying CeO_2_ concentrations (0–4 wt.%). As shown in Figure 3, a large quantity of corundum (Al_2_O_3_, PDF#46-1212) and small amounts of anorthite (CaAl_2_Si_2_O_8_, PDF#41-1486) were detected in the specimens without CeO_2_. As the CeO_2_ content increased from 0 to 2 wt.%, the corundum peak intensities weakened, and the mullite and anorthite peak intensities strengthened. Among them, changes in the mullite phase peak intensities were the most prominent. When CeO_2_ content increased from 2 to 4 wt.%, all the intensities of the mullite, anorthite, and corundum peaks weakened. According to the literature, the Gibbs free energy of (CaO·Al_2_O_3_·2SiO_2_ → 3Al_2_O_3_·2SiO_2_) at elevated temperatures is negative, indicating that calcium feldspar generation of mullite is possible [30]. Therefore, we hypothesize that the presence of CeO_2_ facilitates the conversion of calcium feldspar to mullite. Thus, the peak of the calcium feldspar phase weakens with increasing CeO_2_ content. The addition of excess CeO_2_ led to the formation numerous liquid phases, which existed as glassy phases after cooling. The glassy phases covered the surface of the mullite whiskers, thus weakening the mullite and corundum phase peaks [31].

**Figure 4:**
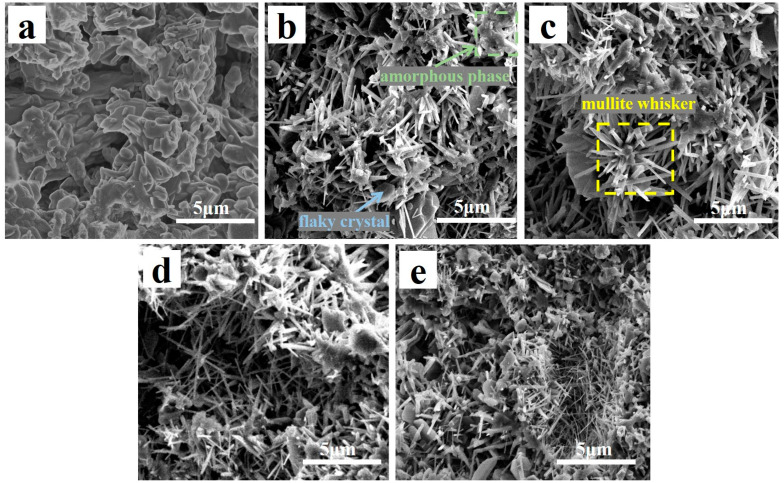
Fracture surface scanning electron microscopy images of porous ceramics with different concentrations of CeO_2_: (**a**) 0 wt.%, (**b**) 1 wt.%, (**c**) 2 wt.%, (**d**) 3 wt.%, and (**e**) 4 wt.%.

Figure 4 shows the SEM images with different CeO_2_ contents (0–4 wt.%) of the samples. Almost no whiskers were detected in the specimens without CeO_2_, as illustrated in Figure 4a. The samples with 1 wt.% CeO_2_ exhibited a combination of irregularly shaped crystals, small needle-like structures, and some flaky crystals [32] (Figure 4b). When CeO_2_ content was increased to 2 wt.%, the porous structure for the specimens was almost supported by arranging in or forming fascicular whisker columns, while the flake-like crystals vanished, and a small amount of amorphous material was attached to the surface of the fascicular whisker columns (Figure 4c) [33,34]. When CeO_2_ content was increased to 3 wt.%, the whiskers decreased in size, while the amorphous phase increased (Figure 4d). The quantity and size of whiskers of specimens containing 4 wt.% CeO_2_ decreased, while flaky crystals resurfaced (Figure 4e).

Studies have shown that the flake-like crystals comprise α-Al_2_O_3_ [4], and the whiskers are composed of mullite. Analysis of the SEM images presented in Figure 4 reveals that the introduction of appropriate content of CeO_2_ to the base materials can accelerate the mullitization process. However, an excessive amount of CeO_2_ hindered mullitization, resulting in the formation of a greater proportion of amorphous material. These results are consistent with the XRD results in Figure 3, where the corundum peak intensities decrease, and that of the mullite phases increase with increasing CeO_2_ content within a certain range (0–2 wt.%). When CeO_2_ content increased from 2 to 4 wt.%, the intensities of mullite, anorthite, and corundum peaks were all weakened. The introduction of CeO_2_ into the starting materials promoted the crystallization of mullite grains, which could be attributed to its strongly accelerated reaction bonding process mechanism, and the dissolution–precipitation mechanism [35]. The former mechanism could be detailed as follows: the low Gibbs (0.26 kJ) of the reaction of Al_2_O_3_ [15] makes it challenging to naturally form mullite when combining Al_2_O_3_ and SiO_2_ without activation. The 4f15d16s2 particular atomic structure of Ce made the atom easily lose electrons, and CeO_2_ easily released oxygen. Studies have shown that incorporating CeO_2_ into the starting material can convert lattice defects of oxygen atoms into activated centers, significantly improving the reaction bonding of mullite from Si and Al_2_O_3_ [36]. This change indicates the generation of a low-viscosity Ce-Al-Si-O liquid phase, facilitating species diffusion, accelerating the oxidation of Si metal, and expediting mullite formation. Furthermore, the addition of CeO_2_ reduces the nucleation energy necessary for mullite whisker growth, leading to a rapid decrease in activation energy [8]. Once the mullite nuclei reach a critical size, there is a sharp decline in activation energy, leading to the rapid growth of mullite whiskers [37]. The process involving the dissolution–precipitation mechanism is influenced by the size difference between Ce^4+^ and Al^3+^ ions. Integrating Ce^4+^ into the gaps of the Al_2_O_3_ lattice to create a solid solution is challenging due to its larger radius. Consequently, Ce^4+^ ions accumulated at the boundaries of mullite grains, impeding grain growth and preventing the formation of secondary phases. This accumulation explains why incorporating an appropriate amount of CeO_2_ into the initial materials increases the production of whiskers and accelerates mullite formation. Conversely, an excess of CeO_2_ hinders crystal growth in one direction, thus decreasing the crystal draw ratio and coarsening of mullite whiskers [26]. Thus, effectively regulating the CeO_2_ content is crucial for the successful fabrication of mullite whiskers.

**Figure 5:**
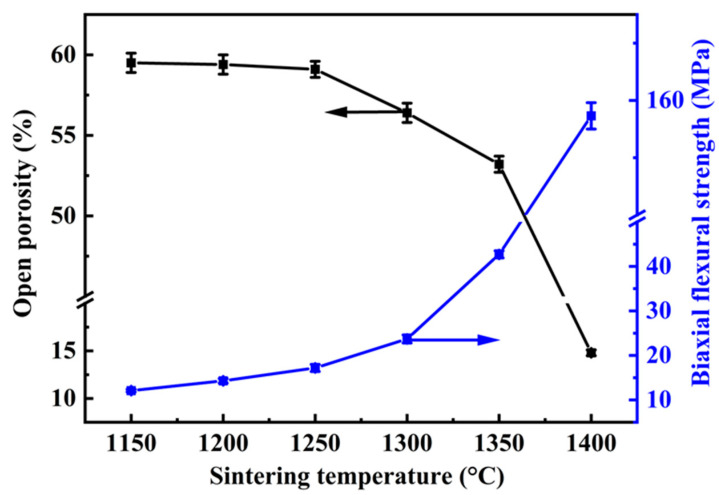
Effect of the sintering temperatures on the open porosity and biaxial flexural strength of specimens with 2 wt.% CeO_2._

Figure 5 shows the effect of the sintering temperatures (1150–1400 °C) on the open porosity and biaxial flexural strength of the specimens of A12C2. Increasing the sintering temperature led to a reduction in the open porosity of the specimens, whereas the biaxial flexural strength increased. When the sintering temperature was relatively low (1150–1200 °C), the open porosity and biaxial flexural strength of the specimens slightly changed. Conversely, elevating the sintering temperature from 1300 to 1400 °C resulted in a noticeable change in both the open porosity and biaxial flexural strength of the specimens. When the sintering temperature was increased from 1150 to 1200 °C, the open porosity slightly decreased from 59.5 ± 0.6% to 59.1 ± 0.5%, while the biaxial flexural strength increased from 12.1 ± 0.3 MPa to 19.2 ± 0.8 MPa. When the sintering temperature was increased from 1300 to 1400 °C, the open porosity decreased from 56.4 ± 0.6% to 14.8 ± 0.3%, whereas the biaxial flexural strength significantly increased from 23.7 ± 0.9 MPa to 157.3 ± 2.3 MPa.

**Figure 6:**
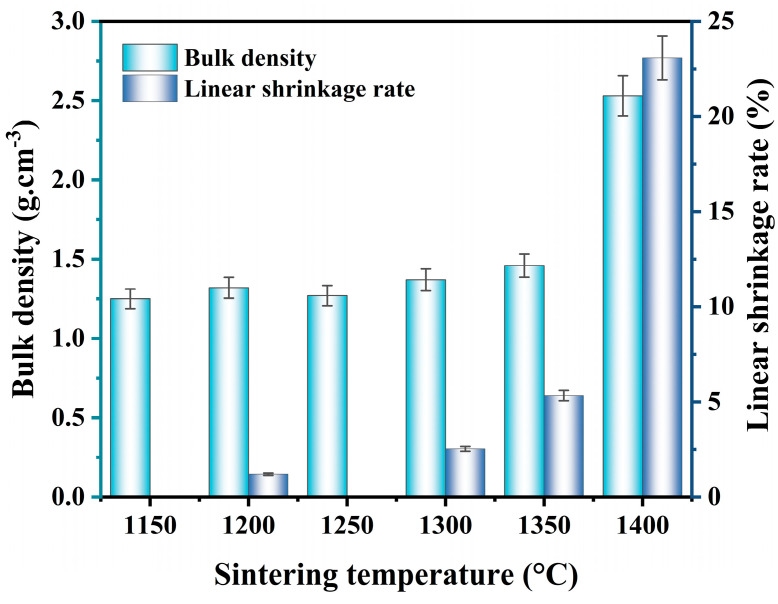
Effect of the sintering temperatures on bulk density of the specimens with 12 wt.% AlF_3_ and 2 wt.% CeO_2_.

Figure 6 illustrates the trend of linear shrinkage rate and bulk density of the sample A12C2, sintered at different temperatures (1150–1400 °C). The bulk density and linear shrinkage of the specimens exhibited similar trends, corresponding to the sintering temperature. They both maintained a consistent level within the sintering temperature range of 1150–1250 °C. The low-temperature sintering was insufficient to form a good bond between the ceramic particles, leading to the existence of more voids and pores inside the ceramics, which reduced the bulk density and linear shrinkage. When the sintering temperature was increased from 1250 to 1400 °C, the bulk density and linear shrinkage of the specimens gradually increased, and the increase was more significant in the relatively high sintering temperature range (1350–1400 °C). When the sintering temperature was increased from 1150 to 1350 °C, the bulk density of the specimens increased from 1.25 g·cm^−3^ to 1.46 g·cm^−3^, and the linear shrinkage increased from 0% to 5.33%. When the temperature was increased to 1400 °C, the bulk density and linear shrinkage increased to 2.53 g·cm^−3^ and 23.07%, respectively. High sintering temperatures induced the formation of glassy phases and accelerated inter-particle bonding in porous ceramics, and the pores in the ceramic materials were further filled. As a result, the overall bulk density and linear shrinkage increased.

**Figure 7:**
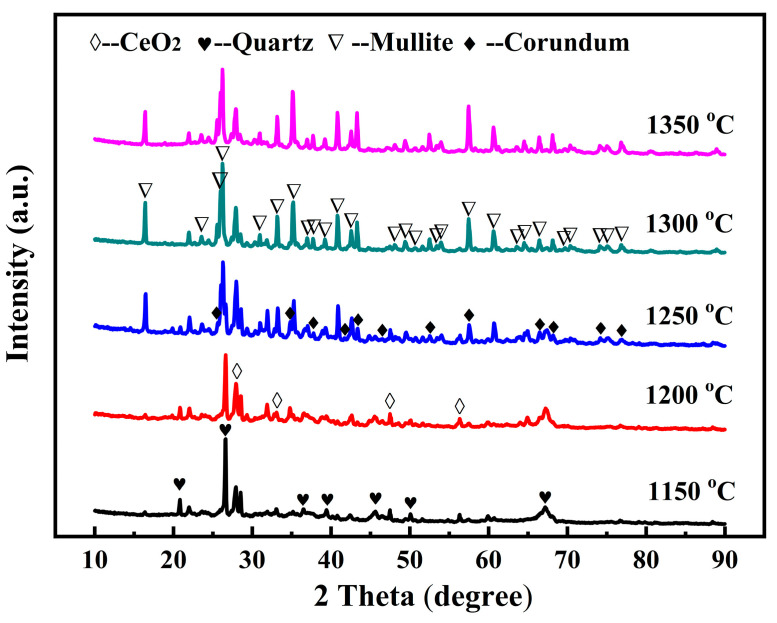
XRD patterns of the specimens of A12C2 sintered at various temperatures for 1 h.

Figure 7 shows the XRD patterns of the specimens of A12C2 sintered at different temperatures (1150–1400 °C). The specimens contained a significant amount of quartz (PDF#46-1045), cerianite (CeO_2_, PDF#43-1002), and a minor amount of albite (NaAlSi_3_O_8_) at 1150 °C. When the sintering temperature was increased to 1200 °C, the quartz peaks diminished, while the albite phase intensities progressively increased, with minimal alterations observed in the cerianite phase peaks. In addition, peaks in the corundum and mullite phases were identified. When the sintering temperature was increased to 1250 °C, anorthite phase peaks appeared, mullite, corundum, and albite peaks became more distinct, and quartz and cerianite peak intensities weakened. At 1300 °C, the mullite and corundum peak intensities significantly increased, and the anorthite peaks slightly changed; however, the albite peaks decreased, and the quartz and cerianite phase peaks disappeared. This phenomenon was attributable to the direct involvement of both the quartz and cerianite phases in the growth of mullite. The albite phase was decomposed to a silicon source or alumina sources, and was indirectly involved in the growth of mullite at a relatively high sintering temperature (1200–1300 °C). When the sintering temperature was increased to 1350 °C, the intensities of the corundum peaks continually increased, while the intensities of all other phase peaks decreased.

**Figure 8:**
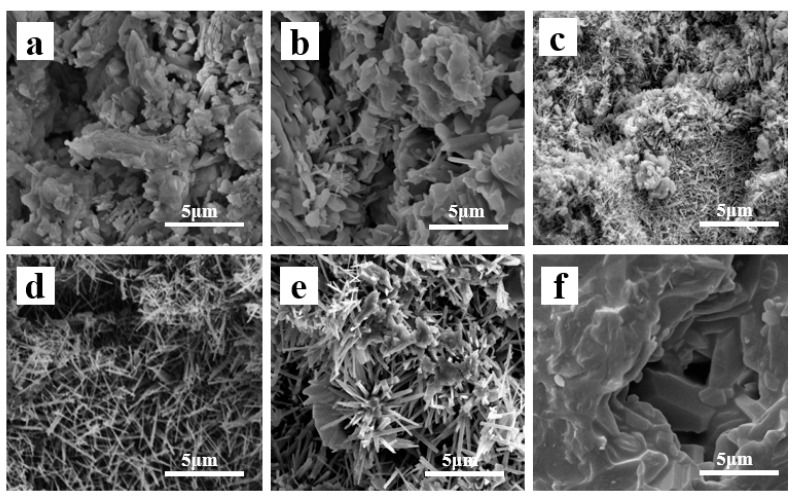
Fracture surface SEM images of the porous ceramics with 12 wt.% AlF_3_ and 2 wt.% CeO_2_ at various temperatures: (**a**) 1150 °C, (**b**) 1200 °C, (**c**) 1250 °C, (**d**) 1300 °C, (**e**) 1350 °C, and (**f**) 1400 °C.

Figure 8 shows the SEM images of A12C2 specimens sintered at different temperatures. Randomly shaped blocks were observed in the specimens sintered at 1150 °C (Figure 8a) without patches or whiskers. Some flake-like crystals and whiskers emerged in the specimens sintered at 1200 °C, with irregularly shaped blocks supporting the porous structure (Figure 8b). The XRD patterns of the specimens sintered at 1200 °C (Figure 7) showed peaks of both the mullite and corundum phase, confirming the whiskers’ crystals were mullite phase, and most of the flake-like crystals were α-Al_2_O_3_. When the sintering temperature was increased to 1250 °C, more needle-like whiskers grew, and only a small amount of the patches and glass-like crystals existed (Figure 8c). When the sintering temperature increased to 1300 °C, the flake-like crystals and the glass phases disappeared, and the porous structure was almost entirely composed of grown needle-like whiskers (Figure 8d). When the sintering temperature increased to 1350 °C, the whiskers coarsened to fascicular whisker columns, and the surface of the fascicular whisker columns was wrapped by the glass phase (Figure 8e). After the sintering temperature was increased to 1400 °C, the whiskers’ porous structure hardened to bulk (Figure 8f), which is probably due to the growth the glass phase had surrounding the whiskers, thus filling the porous structure. Thus, the open porosities rapidly decreased, while the biaxial flexural strength rapidly increased (Figure 5).

Compared to the additives of MoO_3_ used in the previous work, CeO_2_, used in this work, is more effective in forming mullite columns, which enhances the bending strength of samples. Meanwhile, the addition of CeO_2_ also effectively decreased the sintering temperature for the samples, and decreased the grown temperature of mullite whiskers. In this work, with 2 wt.% CeO_2_, the mullite whiskers were detected in the samples sintered at 1200 °C, which is 100 °C lower than that of the previous work. Moreover, the sintering temperature for the full growth of the mullite whiskers in the porous structure was 1300 °C, which is 50 °C lower than that of the previous work. Consequently, the specimens sintered at 1350 °C exhibited excellent properties, including an open porosity of 53.2 ± 0.5% and a biaxial flexural strength of 42.7 ± 0.8 MPa. Furthermore, the mullite whiskers in the specimen were uniformly distributed and clustered, similar to a hedgehog, with a diameter of 0.05–0.5 µm, length of 8–10 µm, and aspect ratios (length to diameter ratio) of 10–20, on average.

**Figure 9:**
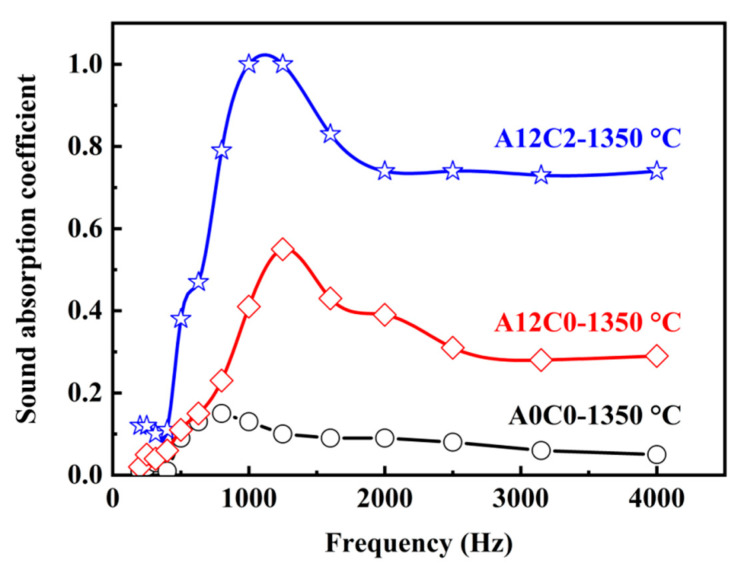
Effect of the content of AlF_3_ and CeO_2_ on the sound absorption coefficient of the specimens.

Figure 9 shows a peak of sound absorption in the low-frequency range between 630 and 2500 Hz. In the medium-frequency range, the sound absorption coefficient slightly decreased and then stayed at the same level with increasing frequency. The specimens with 12 wt.% AlF_3_ and 2 wt.% CeO_2_ had the highest sound absorption coefficients. The presence of lattice defects in CeO_2_ facilitated the release of oxygen atoms toward the activation center, resulting in the formation of a low-viscosity Ce-Al-Si-O liquid phase [38]. This liquid phase enhanced material diffusion and accelerated the creation of mullite whiskers. Consequently, porous ceramics exhibited a greater number of gas–solid interfaces, promoting enhanced reflection, scattering, and friction between sound waves and pore walls. This transformation led to the more efficient conversion of sound energy into heat energy [39]. Additionally, the even dispersion of whiskers across the surface of specimens contributed to increased surface roughness of the ceramic, further amplifying friction and reducing air viscosity [40].

**Figure 10:**
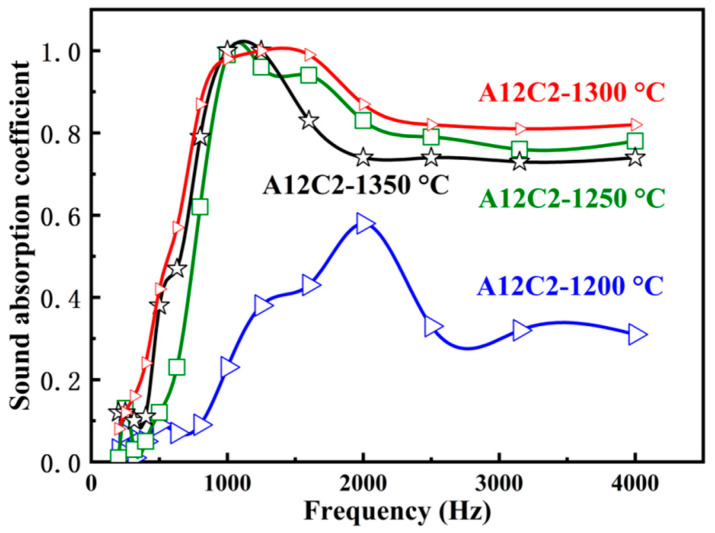
Effect of the sintering temperatures on sound absorption coefficient of the specimens.

Similar to Figure 9, all the curves of specimens in Figure 10 present the sound absorption coefficients of the A12C2 specimens sintered at 1200–1350 °C in the frequency range from 200 to 4000 Hz. Figure 10 shows a peak of sound absorption in the low-frequency range (200–2500 Hz), but then slightly decreased and remained stable at relatively higher frequencies. The sound absorption coefficient of the specimens sintered at 1250–1350 °C was higher than the specimen sintered at 1200 °C. The specimens sintered at 1300 °C had excellent sound absorption properties, i.e., a sound absorption coefficient of ≥ 0.98 between 1000 and 1600 Hz, and ≥ 0.80 between 2500 and 4000 Hz. A comparison of the data from this work with data reported in the literature is shown in Table 1.

**Figure 11:**
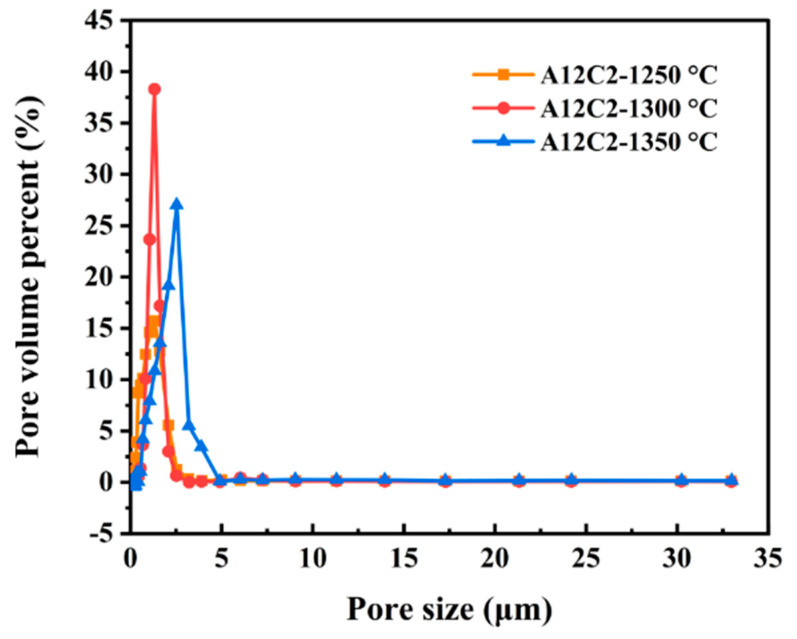
Effect of the sintering temperatures on the pore size distribution of the specimens.

Figure 11 shows the pore size distribution of sample A12C2 at different sintering temperatures. The average pore size distributions of the samples at 1250, 1300, and 1350 °C were all single-peaked, and they were 1.32, 1.32, and 2.54 μm, respectively, and the corresponding cumulative volume fractions were 15.7, 38.3, and 27.0%, respectively. Figure 11 shows that reducing the average pore size improved the acoustic absorption properties of the ceramic samples. When sound waves propagated to the surface of porous ceramics and entered the pores, they triggered the vibration of the air inside the pores. If the pore size is relatively small, the vibration is more likely to form a resonance effect. Resonance can more effectively convert acoustic energy into thermal energy, thus consuming the energy of the sound wave to achieve the effect of sound absorption. The pore structure of porous ceramics is regarded as a series of tiny Helmholtz resonators. As the pore size decreased, the area of the radial opening of the Helmholtz resonator also decreased, lowering the resonance frequency of the resonator. Therefore, as the pore size decreased, the absorption peak of porous ceramics moved toward lower frequencies, thus broadening the sound absorption frequency range. When sound waves propagated in smaller pore diameters, the energy of the sound waves was rapidly converted into heat energy due to the friction of the pore walls and the viscous resistance of the air [44].

**Figure 12:**
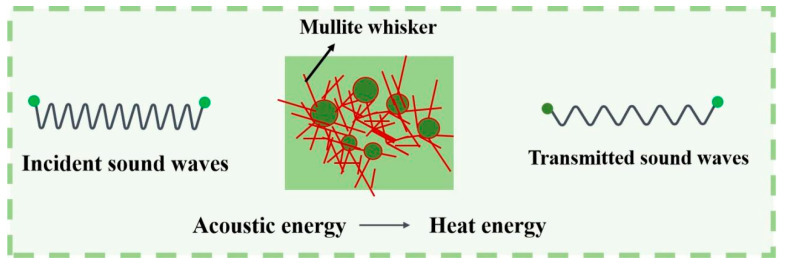
Demonstration diagram of sound absorption and noise reduction of porous ceramics.

In the frequency range of 1000–1600 Hz, the longer wavelength of the sound waves led to increased diffraction and scattering as the waves propagated through the porous ceramic. This process, combined with the friction between air molecules and the pore walls, led to the conversion of sound energy into internal energy or other forms of energy, improving sound absorption. Reducing the pore size of the porous ceramics enhanced the reflection of sound waves within the material, increasing the vibration of air molecules and the friction between these molecules and the pore walls [45,46]. However, from 2500 to 4000 Hz, the shorter wavelength of the sound waves implied that simple channels might not effectively capture and attenuate the high-frequency sound waves. If the pore size is relatively large compared to the high-frequency sound waves, the sound waves may be reflected inside the pores rather than being absorbed. This reflection would consequently diminish the sound absorption effectiveness. The more pores inside the porous ceramics increased the reflection, scattering, and friction between the sound waves and the pore walls. Therefore, more acoustic energy was converted into thermal energy (Figure 12). The open porosity of the sintered specimens at 1300 ℃ was slightly lower than that of the sintered specimens at 1250 °C and 1200 °C (Figure 4). However, the acoustic absorption performance of the sintered specimens was better at 1300 ℃ than the sintered specimens at 1200 °C. This improved performance was attributed to the numerous micropores and microcracks in the sintered porous ceramic specimens at 1300 °C. The presence of micropores and microcracks increased friction and viscous air depletion, which improved the acoustic absorption performance [40].

## 3. Experimental

### 3.1. Raw Materials for Porous Ceramics

The raw materials for this study were construction waste, Al_2_O_3_, AlF_3_·3H_2_O, and CeO_2_. Construction waste was sourced from Guangzhou Shizheng Environment Co., Ltd. in Guangdong Province, China. This raw material underwent dry ball milling in a planetary ball mill (QM-ISP4-CL, Instrument Plant of Nanjing University. Dimensions: 570 × 380 × 450 mm, 100 mL corundum ball mill can). The sizes of zirconia ball milling beads were 1 mm^3^, 5 mm^3^, and 10 mm^3^, and the ratio of ball milling beads of each size was 3:5:2. The milling process was conducted at 300 rpm for 1 h, and sieved through a 0.6 mm sieve. The chemical composition of the construction waste was determined using the silicate chemical composition rapid analyzer (GKF-IV, Xiangtan Xiangyi Instrument Co., Ltd., Xiangtan, China). As shown in Table 2, the chemical compositions are 71.65 wt.% SiO_2_ and 7.67 wt.% Al_2_O_3_.

### 3.2. Fabrication of Porous Ceramics

Al_2_O_3_ powder (AR, Chinasun Specidalty Products Co., Ltd., Changshu, China) was used as the alumina source (presented as 3Al_2_O_3_·2SiO_2_) to produce the mullite reinforcement due to the inadequate amounts of aluminum in the construction waste. AlF_3_·3H_2_O (98.0–102.0%, Sinopharm Chemical Reagent Co., Ltd., Shanghai, China) and CeO_2_ (99.99%, Sinopharm Chemical Reagent Co., Ltd., Shanghai, China) were used as the crystallization catalyst and sintering aids, respectively. AlF_3_ played the following dual roles: promoting mullite whisker formation (by transforming to Al_2_O_3_ and the subsequent reaction with SiO_2_) and serving as a partial aluminum source for forming stoichiometric 3:2 mullite. In the Al_2_O_3_-SiO_2_-AlF_3_ system, the chemical process to generate mullite proceeded as follows [47]:

(1) $${6AlF}_{3}\left(s\right)+3O_{2}\to 6AlOF\left(g\right)+12F\left(g\right)$$

(2) $${Al}_{2}O_{3}\left(s\right)+2F\left(g\right)\to 2AlOF\left(g\right)+0.5O_{2}\left(g\right)$$

(3) $$2{SiO}_{2}\left(s\right)+8F\left(g\right)\to 2{SiF}_{4}\left(g\right)+2O_{2}\left(g\right)$$

(4) $$6AlOF\left(g\right)+2{SiF}_{4}\left(g\right)+3.5O_{2}\left(g\right)\to 3{Al}_{2}O_{3}\cdot 2{SiO}_{2}\left(s\right)+14F\left(g\right)$$

A series of porous ceramics were synthesized using the stoichiometric mullite composition with varying AlF_3_ and CeO_2_ content. In this study, these ceramics were designated as A*x*C*y*, where A represents AlF_3_, C represents CeO_2_, and *x* and *y* indicate the respective mass percentages of each compound in the raw materials of the samples. Detailed compositions are provided in Table 3.

### 3.3. Characterization and Test

The phases of the construction waste and the sintered specimens were analyzed via powder X-ray diffraction (XRD; X’Pert Pro, PANalytical Co., Almelo, Holland) with Cu Kα radiation (λ = 0.15418 nm), using graphite monochromatization. Operating parameters included a tube voltage of 40 kV, a tube current of 40 mA, a 2θ angle step size of 0.0330°, a scan step time of 10.160 s, and a 2θ angle scan range from 10° to 90°. The open porosity and bulk density of the sintered specimens (which corresponded to rectangular green pieces measuring 5 mm × 20 mm × 48 mm) were determined using Archimedes’ method as per ASTMC 20-92, using distilled water as the immersion medium. Each specimen underwent ten parallel measurements, with each measurement repeated five times, and the average result was recorded as the measured value of the specimen.

The biaxial flexural strength of the sintered specimens was evaluated using an electronic digital control system (INSTRON-5567, Co. Instron Engineering Corporation, Boston, USA) through the three-point bending test [35,36], conducted at room temperature. Standardized specimens (3 mm × 4 mm × 35 mm) were polished and subjected to a 30 mm span at a loading rate of 0.5 mm/min. Each specimen was tested in eight parallels, and the average measurement results were recorded. The microstructures of the sintered specimens were examined using scanning electron microscopy (Nova Nano SEM 430, FEI, Hillsboro, OR, USA). The sound absorption coefficient of the sintered specimens (100 mm × 50 mm × 10 mm) was determined. The 100 mm diameter specimen was used to assess sound absorption coefficients for lower frequencies (200–2000 Hz), while the 50 mm diameter specimen was used for higher frequencies (2500–4000 Hz). The test was conducted using the JTZB-type system (Beijing Century Jiantong Technology Development Limited Company, Beijing, China) and the standing wave tube method, following ASTM E 1050-98. Additionally, the transfer function methodology was used as specified in the EN ISO 10534-2 standard. The sound absorption coefficient (α) was calculated using the following Equations (5) and (6):

(5) $$\alpha =\frac{4n}{{\left(1+n\right)}^{2}}$$

(6) $$n=\frac{\left|p_{max}\right|}{\left|p_{min}\right|}$$

where *n* is the standing wave ratio, and *p*_max_ and *p*_min_ are the maximum and minimum sound pressures in the impedance tube, respectively.

The ceramic sample line shrinkage *S*_0_ (%) is calculated as follows in Equation (7):

(7) $$S_{0}=\frac{L_{0}-L}{L_{0}}$$

where *L*_0_ is the sample length before sintering, and *L* is the sample length after sintering.

## 4. Conclusions

In situ mullite whisker-reinforced porous sound-absorbing ceramics with high strength were successfully prepared with construction waste and Al_2_O_3_ powder as the raw materials, and AlF_3_ and CeO_2_ as the additives and mineralizing agents, respectively. A small amount of CeO_2_ effectively enhanced the growth of elongated mullite crystals in the porous ceramics, decreased the growth temperature of the mullite whiskers, and increased the biaxial flexural strength of the specimen, whereas excessive CeO_2_ inhibited the mullitization. By co-adding 2 wt.% CeO_2_ and 12 wt.% AlF_3_ in the system, mullite whiskers were successfully obtained at a sintering temperature of 1300 °C for 1 h. The obtained mullite whiskers exhibited excellent properties, including an open porosity of 56.4 ± 0.6%, an average pore size of 1.32–2.54 μm, a biaxial flexural strength of 23.7 ± 0.9 MPa, and a sound absorption coefficient of > 0.8 at 800–4000 Hz. This work offers a cost-effective method for the future preparation of high-performance, porous, sound-absorbing ceramics from construction waste.

## Figures and Tables

**Table 1:** Performance comparison of porous sound-absorbing ceramics.

The Main Raw Material	Porosity (%)	Compressive Strength (MPa)	Frequency Range (Hz)	Sound Absorption Coefficient
Al_2_O_3_, ZrO_2_ [41]	97.8–98.0	0.066–0.091	500–6500	0.60–0.77
SiC [42]	76–82	1.21–5.13	200–1600	<0.80
Na_2_B_4_O_7_·10H_2_O [3]	-	1.12 ± 0.05	200–1400	<0.58
Al_2_O_3_/mullite [43]	77–82%	0.27–0.68	50–1400	0.30–0.99
This word	56.4 ± 0.6	-	800–4000	>0.80

**Table 2:** Main chemical composition (wt.%) of the construction waste.

Materials	Chemical Composition (wt.%)								
	LI	SiO_2_	Al_2_O_3_	Fe_2_O_3_	TiO_2_	CaO	MgO	K_2_O	Na_2_O
Construction waste	6.36	71.65	7.67	1.71	0.21	9.20	0.62	1.97	0.79

Note: LI stands for loss of ignition.

**Table 3:** Mixing ratio of raw materials.

Sample ID	Construction Waste (wt.%)	Content of Al_2_O_3_ (wt.%)	Content of CeO_2_ (wt.%)	Content of AlF_3_ (wt.%)
A0C0	29.08	70.92	0	0
A12C0	25.59	62.41	0	12
A12C1	25.30	61.70	1	12
A12C2	25.01	60.99	2	12
A12C3	24.72	60.28	3	12
A12C4	24.43	59.57	4	12

## Data Availability

The original contributions presented in the study are included in the article, further inquiries can be directed to the corresponding author/s.
